# Long-term exposure to PM_2.5_ and cardiovascular disease incidence and mortality in an Eastern Mediterranean country: findings based on a 15-year cohort study

**DOI:** 10.1186/s12940-021-00797-w

**Published:** 2021-10-28

**Authors:** Soheila Jalali, Mojgan Karbakhsh, Mehdi Momeni, Marzieh Taheri, Saeid Amini, Marjan Mansourian, Nizal Sarrafzadegan

**Affiliations:** 1 Student Research Committee, Department of Epidemiology and Biostatistics, School of Health, Isfahan University of Medical Sciences, Isfahan, Iran; 2 Department of Community and Preventive Medicine, School of Medicine, Tehran University of Medical Sciences, Tehran, Iran; 3 Department of Surveying Engineering, University of Isfahan, Isfahan, Iran; 4 Pediatric Research Center, Cardiovascular Research Institute, Isfahan University of Medical Sciences, Isfahan, Iran; 5 Department of Surveying and Geomatics Engineering, University of Isfahan, Isfahan, Iran; 6 Isfahan Cardiovascular Research Center, Cardiovascular Research Institute, Isfahan University of Medical Sciences, Isfahan, Iran; 7 Department of Automatic Control, Biomedical Engineering Research Center, Universitat Politècnica de Catalunya, BarcelonaTech (UPC), Building H, Floor 4, Av. Diagonal 647, 08028 Barcelona, Spain; 8 School of Population & Public Health, University of British Columbia, Vancouver, Canada

**Keywords:** PM_2.5_, Outdoor air pollution, Mortality, Cardiovascular diseases, AMI, Stroke, Survival models, Cox proportional hazards frailty models

## Abstract

**Background:**

Evidence concerning the impact of long-term exposure to fine Particulate Matter ≤2.5 μm (PM_2.5_) on Cardio-Vascular Diseases (CVDs) for those people subject to ambient air pollution in developing countries remains relatively scant. This study assessed the relationship of 15-year PM_2.5_ exposure with cardiovascular incidence and mortality rate in Isfahan province, Iran.

**Methods:**

The cohort comprised 3081 participants over 35 years old who were free of CVDs. They were selected through multi-stage cluster sampling in Isfahan, Iran. PM_2.5_ exposure was determined separately for each individual via satellite-based spatiotemporal estimates according to their residential addresses. In this context, CVD is defined as either fatal and non-fatal Acute Myocardial Infarctions (AMI) or stroke and sudden cardiac death. The incidence risk for CVD and the ensuing mortality was calculated based on the average PM_2.5_ exposure within a study period of 15 years using the Cox proportional hazards frailty model upon adjusting individual risk factors. The mean annual rate of PM_2.5_ and the follow-up data of each residential area were combined.

**Results:**

Mean three-year PM_2·5_ exposure for the cohort was measured at 45.28 μg/m^3^, ranging from 20.01 to 69.80 μg/m^3^. The median time period for conducting necessary follow-ups was 12.3 years for the whole population. Notably, 105 cardiovascular and 241 all-cause deaths occurred among 393,786 person-months (27 and 61 per 100,000 person-months, respectively). In well-adjusted models, 10 μg/m^3^ increase in PM_2.5_ corresponded to a 3% increase in the incidence rate of CVDs [0.95 CI = 1.016, 1.036] (in case of *p* = 0.000001 per 10 μg/m^3^ increase in PM_2.5_, the Hazard Ratio (HR) for AMI and Ischemic Heart Disease (IHD) was 1.031 [0.95 CI = 1.005, 1.057] and 1.028 [0.95 CI = 1.017, 1.039]), respectively. No consistent association was observed between PM_2.5_ concentration and fatal CVD (fatal AMI, fatal stroke, SCD (Sudden Cardiac Death)) and all-cause mortality.

**Conclusions:**

Results from analyses suggest that the effect of PM_2.5_ on cardiovascular disease occurrence was stronger in the case of older people, smokers, and those with high blood pressure and diabetes. The final results revealed that long-term exposure to ambient PM_2.5_ with high concentrations positively correlated with IHD incidence and its major subtypes, except for mortality. The outcome accentuates the need for better air quality in many countries.

**Supplementary Information:**

The online version contains supplementary material available at 10.1186/s12940-021-00797-w.

## Background

The current concentration of ambient air pollution may cause detrimental health effects including a high rate of mortality and morbidity, partly induced by CVD. Based on epidemiological findings specific to western countries, elevated particles with a mass median aerodynamic diameter ≤ 2.5 μm (PM_2.5_) consistently lead to CVD incidence and death [1]. The fine particulate matter calculated as 2.5 μg/m^3^ or less is in close association with myocardial infarctions, thrombosis, and stroke [2]. The mean of air pollutant concentrations in the USA [3] and European [4] countries is much lower than that in Eastern Asia [5]. It should be noted that Eastern Asia is usually bombarded with frequent Asian dust events from industrial facilities and the Gobi Desert in Central Asia, reportedly producing over 20% of the total global dust emissions through the westerlies [6, 7].

The dominance of vast deserts in the surrounding areas of Isfahan, Iran together with the significant number of large industries in its suburbs constitute the reasons why the city is one of the most polluted cities in Iran [8]. High PM_2.5_ levels in Isfahan could be arguably attributed to the growing emergence of deserts and mines, of lead and zinc, as well as energy conversion systems such as power plants and oil refineries in immediate surroundings. In addition, in Isfahan province, wind direction changes per season, meaning that with the exception of summer, the winds generally blow from the west to the east. A significant impact on the air quality was observed in Isfahan which inevitably resulted from the adaptation of wind currents to the degraded areas caused by the activities of gypsum, clay, sand, and mines as well as a wide range of related industries in more than 12,900 ha (6800 ha of gypsum and 6100 ha of clay and sand). The concentration of suspended particles in the air was the key to the process. Furthermore, studies that have investigated the amount of dust and suspended particles in Isfahan’s main stations suggest that the operations of local centers have yielded more than 30% increase in dust volume in the summer. As mentioned above, such particles are produced by summer winds in the east [9].

Over 90% of the world’s population inhabit areas whose air quality exceeds the mean annual PM_2.5_ standard defined at 10 μg/m^3^ by the World Health Organization (WHO) [10].

Furthermore, such concentration-responsive detrimental impacts of long-term PM_2.5_ exposure on cardiovascular mortality rates in countries with higher air pollution levels have not been thoroughly investigated. Therefore, it is crucial to carefully scrutinize the effects of such exposure and develop fitting preventive strategies to reduce potential risks for the community. Given the accessibility of data about individual exposure to air pollution and the significance of other individual CVD risk factors, the stated problem has been addressed in the present study.

Assessments carried out in line with the Global Burden of Disease initiative demonstrate that high systolic blood pressure, smoking, high-sodium diet, and ambient particulate matter pollution are among the leading four risk factors contributing to deaths and disability-adjusted life years across the world [11].

Nearly 50% of these estimated attributable deaths result from ischemic heart disease and stroke, to which countries with low and medium incomes and high outdoor PM_2·5_ levels are primarily subject [12].

Most air pollution studies enjoying a large cohort did not make appropriate adjustments for individual-level risk factors (e.g., Social Economic Status (SES), health-related behaviors, or healthcare access) to the extent that they failed to properly appreciate the associations between PM_2·5_ and CVD.

The aging population and fast-paced urbanization are increasingly adding fuel to the prevalence of cardiovascular diseases in Iran.

Statistics retrieved from the Iranian Journal of Cardiovascular Nursing point to the cardiovascular diseases (incident cases) as the most frequent cause of disability in most countries including Iran in 2013 [13]. To be specific, the total number of cardiovascular diseases in the present study was 441, with 351 cases (79.6%) in urban and 90 (20.4%) in rural areas and it followed a steadily rising trend.

A precise assessment of PM_2.5_ exposure could be quite beneficial to medical studies with the help of satellite data. Satellite observations measure the Earth’s optical radiations affected by aerosols. There are physical and acceptable statistical models for calculating PM_2.5_ density via satellite observations [14].

Backed by the datasets obtained from high-quality satellite-based PM_2.5_ estimates with a 1 × 1 km spatial resolution as well as the prediction records of Cardiovascular Disease Risk in Iran (Isfahan Cohort Study (ICS) - an established prospective project), the present study attempts to discover the potential relationship between long-term PM_2.5_ exposure and CVD-induced mortality and morbidity with respect to total CVD and its subtypes. This is reportedly the first study that has investigated the longitudinal effect of PM_2.5_ on cardiovascular diseases in Iran. In addition, it investigates the possibility that PM_2.5_ greater than a specific value (PM_2.5_ > 56.61) be closely associated with increased risks of CVD-induced morbidity and mortality. Subgroup analysis was carried out to study the PM_2.5_-induced morbidity and mortality under cardiovascular subtypes.

It is hoped that the findings of the present study would enhance conventional understandings about the global impacts of PM_2·5_-induced air pollution on CVDs given that they capture different population communities subject to a wide range of PM_2·5_ concentrations on a global scale and include standardized objective measures for CVD risk factors.

## Materials and methods

### Study population

Isfahan Cohort Study (ICS) was employed to investigate CVD incidence and the corresponding risk factors involved in the case of Iranian population. ICS is a population-based, longitudinal study that targets 6504 adults aging equal to or greater than 35 years at baseline, living in urban and rural areas from three counties in central Iran (Isfahan, Arak, and Najafabad) who had participated in the baseline survey of a community trial for CVD prevention and control, entitled Isfahan Healthy Heart Program (IHHP). They were recruited from January 2 to September 28, 2001. The baseline survey of the IHHP was conducted in a representative population of adults aging ≥19 years who were living in urban and rural areas of Isfahan, Arak, and Najafabad. ICS comprised 3081 adults living in 37 clusters. The participant selection method was multi-stage random sampling. The study population was first classified in terms of participants’ area of residence (urban or rural), and the regional population distribution was obtained from the national population census conducted in 1999. Census blocks were randomly selected from each county with a probability of selection proportional to the expected number of households, which were divided into approximately 1000 households. In each cluster, approximately 5-10% of the households were randomly selected for enumeration. One eligible 19-year-old subject was randomly selected from each house, provided they were mentally stable, unpregnant, and Iranian in nationality. The response rate for home interviews was 98%. However, only 95% attended the examination clinic. Because individual cardiovascular health was not included in the IHHP sampling criteria, participants initially included 108 cases (2.8) with a history of MI, stroke, or heart failure who were excluded from the ICS survey. IHHP samples were derived among different age and sex groups to reflect the fair age-sex distribution of the community. The prevalence of cardiovascular risk factors in the control area was estimated to be 0.2, and the sample size for IHHP had 90% strength to detect a relative risk of 0.75 at a significance level of 0.05. Arranged in the form of clustering, the total sample size in each region comprised 4828 people. To perform a series of follow-ups concerning the losses, we managed to employ 6300 people from each region (12,600 people). A total of 12,514 people were included in the initial survey. Individual selection for ICS ethical certification was obtained from the ethics committee of the Isfahan Cardiovascular Research Center, i.e., a cooperative center at World Health Organization. Of the 12,514 individuals reviewed in the baseline survey, the total number of 6640 adults aging ≥35 years were enrolled into the ICS [15]. A full and detailed description of the study design was already given in [15].

Given that our study solely focuses on Isfahan province, the samples from Isfahan and Najafabad were included in the analysis, only. A total of 3081 adults from both rural and urban areas with complete information were initially enrolled for baseline examinations in 2001.

Multi-stage cluster random sampling was employed to select participants in terms of gender, age, and residential status (i.e., urban or rural). By using a formula for calculating the sample size, 3081 of them were selected in 2001.

Following an initial review of samples over the years 2001 to 2016, the follow-up procedure (telephone interview) for the candidates was performed every 2 years. Initial structured interviews were conducted based on a questionnaire with three main questions. “Is he/she alive?”, “has he/she been hospitalized for any reason? (With special focus on cardiovascular and cerebrovascular events), and “did the participant experience any of the following five neurological symptoms (hemiparesis, dysarthria, facial asymmetry, imbalance, and transient intermittent blindness)?” In the event of death, hospitalization, or neurological symptoms, the date of the event and the physician’s diagnosis must be taken into account. In case of any event, the questionnaire was reviewed along with relevant health records. In the case of out-of-hospital deaths, death certificates from the provincial mortality database were obtained and a verbal autopsy was performed by a specialist nurse over the course of a secondary interview in the presence of the surviving family members. A pre-defined questionnaire composed of such matters as medical history, signs, and symptoms of the patient prior to his/her death was made use of in the verbal autopsy [15].

The rate of ICS loss in the follow-up process was 6.4 and 3.9% in the second and third phases, respectively. Moreover, Arak province is situated in the central region of northwest of Isfahan and due to such problems as distance and difficulty in taking samples in rural areas, it was removed from the second phase of the ICS. Then, only Isfahan province was considered in this study to achieve complete follow-ups. Details of the loss to follow-up information and other issues are accessible in the following references [16, 17].

### Measurements of the confounding factors

Having signed the informed written consent forms, the participants under study were interviewed by trained health professionals for 30 min. A valid questionnaire consisting of questions about demographic characteristics, socioeconomic statuses, behaviors, attitudes, skills, and knowledge about chronic, non-communicable, and other related diseases was employed. The participants’ lifestyle behaviors (smoking, physical activities, and eating habits), measurement of blood pressure, and anthropometric parameters were taken into consideration in compliance with standard protocols [18, 19] and calibrated instruments were used in this regard [15]. In terms of smoking status, subjects were categorized as smokers (at least one cigarette per day or at least one cigarette per day in the past) and non-smokers [15]. Participants’ fasting blood samples (10 ml) were obtained so as to gauge their total cholesterol (mg/dl), HDL-cholesterol (HDL-C), LDL-cholesterol (LDL-C), and triglycerides (mg/dl). The ratio of LDL to HDL was divided into three categories: normal (< 5.4), borderline (5.4-7), and abnormal (> 7) [20]. Diabetes mellitus is defined as a condition with fasting blood glucose ≥126 mg/dl or when the patient is under anti-diabetic treatment. Impaired glucose tolerance is identified if the 2-h glucose levels are equal to and above 140 and below 200 mg/dl [21].

Obesity, overweight, and normal weight constitute the Body Mass Index (BMI): BMI ≥30 kg/m^2^, 25 ≤ BMI < 29.9 kg/m^2^, and BMI < 24.9 kg/m^2^ [22].

Hypertension was defined as a condition with blood pressure ≥ 140/90 mmHg or when the patients were under anti-hypertensive treatment [23].

A standard Food Frequency Questionnaire (FFQ) was employed to determine the participants’ dietary behaviors with appropriate validity and reliability [24].

Food Frequency Questionnaire includes dishes that are commonly consumed by Iranians. For each food item, participants were asked to report the amount and frequency of consumption in the past year. The total diet score is calculated as the sum of the scores given for each of the 12 food groups. Those subjects with a diet consisting of cereals, hydrogenated vegetable oils, red meat, and processed meat are considered healthy. The unhealthy group consumed sweets and pizza as well. Therefore, the total diet score for each person could range between 0 and 12. Participants with a total diet score of 8-10 are classified as healthy, while people with a diet score below 8 are classified as poor [25].

Physical activity was measured according to the International Physical Activity Questionnaire (IPAQ), the validity and reliability of which had already been reviewed and reported [26–28], and it was divided into three categories: low (less than 600 Met-min/Week), medium (at least 600 Met-min/Week), and high (at least 3000 Met-min/Week) [29].

This research was endorsed by the Isfahan University of Medical Sciences and approved by the Health Sciences Research Ethics Board, the code number for which is: 399099. The written consent forms were signed by each participant prior to data collection.

### Outdoor PM_2·5_ air pollution exposure assessment

A satellite-based spatiotemporal model with a spatial resolution of 1 × 1 km was utilized to measure ambient PM_2.5_ levels. PM_2.5_ modeling refers to Land Use Regression (LUR) models that have recently drawn much attention because of their critical role in understanding and assessing air pollution [14, 30].

MODIS (Moderate Resolution Imaging Spectroradiometer) aerosol products, namely MOD04, and MAIAC (Multi-Angle Implementation of Atmospheric Correction) are multispectral sensors onboard two Terra and Aqua satellites and they were utilized in the present study. The satellites overpass the study area at approximately 10:30 and 13:30 local times every day [31]. The statistical model proposed by Kong et al. [32] was employed to convert the obtained statistical observations to PM_2.5_. Thus, the seasonal localized regression coefficients were recalculated for our area based on the model presented by Kong et al. [32] and suggestions from Li et al. [33].

The data associated with ground stations in the city of Isfahan were taken from the Department of Environment for the reference years 2013 and 2018. Cloudy days, as well as days with wind speeds of more than 10 m/s, were put aside in compliance with the reports of the Iran Meteorological Organization for Isfahan. On such days, instantaneous satellite measurements significantly differ from ground-based measurements on a daily average, an issue that causes uncertainty.

The mean, standard deviation, and maximum PM_2.5_ concentration were calculated, and outlier data were deleted at a confidence level of 99%. In both of our references (Kong et al. [32] and Li et al. [33]), the seasonal division of data yielded better results than annual calculation. The seasons were divided according to those recommendations and based on the local climate (see Additional file 1).

In the method proposed by Kong et al. [32], calculating the average AOD in an area of 50 × 50 km was recommended so as to eliminate the effects of Land uses, although this averaging would make the PM_2.5_ estimations coarse in terms of spatial resolution. However, Li et al. [33] implemented the LUR model to control Land-use effects based on the variables such as population density, distance to industrial areas, and the area slope. Thus, they keep the spatial resolution, especially when considering the area within a city, as in our case.

Then, we use seasonal linear regression functions between AOD and PM_2.5_ which can be represented as PM_2.5_ = a * AOD + b, where a and b are regression coefficients. Regression coefficients are rounded to significant digits according to their estimation error (see Additional file 2).

A three-year rolling average of PM_2·5_ estimates was available in 2001, 2007, and 2013 with an approximate resolution of 1 × 1 km. Patients were enrolled in 2001 and their information was recorded and followed up until the end of 2015. PM_2·5_ levels in the participants’ residences were measured in 2001, 2007, and 2013, with an approximate resolution of 1 × 1 km. The values for the days of each year and subsequently, those for the 3 years were averaged. The mean of PM_2.5_ values for the years before the event was calculated for each participant, i.e., if individuals survived before 2007, only the amount of PM_2.5_ in 2001 was calculated; the same holds for the following: if they survived before 2013, the average PM_2.5_ for the years 2001 and 2007 was assessed; and if they survived before 2015, the average PM_2.5_ for 2001, 2007, and 2013 was measured. Indeed, the estimated exposure for the study participants was the mean concentration of PM_2.5_ over their surviving years during the cohort period.​​ The obtained result was subsequently inputted in the model as a three-year rolling average and descriptive information about the three-year rolling average of PM_2·5_ variable was eventually provided.

### Outcomes

The reported cases of Acute Myocardial Infarction (AMI) and stroke were monitored by the Isfahan Cardiovascular Research Center. In the absence of admission data in the registration database, well-trained nurses reviewed hospital-related medical records. The diagnosis of acute MI was based on the presence of at least two of the following criteria: (a) typical chest pain lasting more than 30 min with ST elevation> 0.1 mV in at least two adjacent electro cardiograph leads and (b) increased serum levels of cardiac biomarkers [34, 35]. Heart death was defined as death within 1 h of disease onset, cardiac arrest, or sudden collapse exhibiting no symptoms 41 h in advance. Ischemic Heart Disease (IHD) includes definite or probable MI, Unstable Angina (UA), and sudden cardiac death. In addition, the definition of stroke employed in this study to help diagnose stroke is in line with the definition presented by the World Health Organization. CVD was perceived as a combination of IHD and stroke. Although the doctors’ diagnoses were taken into account in the hospital, final decisions were made independently by the board [15]. Total CVD, fatal CVD, non-fatal CVD, all-cause mortality, stroke, AMI, and IHD were separately included in different models.

### Statistical analysis

Description of the baseline characteristics of the participants was issued based on means, standard deviation for continuous measurements, and percentages of categorical variables. The present study used the frailty model as an extended form of multivariate-adjusted Cox proportional hazards to estimate Hazard Ratios (HRs) and Confidence Interval of 95% (CIs) in the case of incidence of total CVD and its subtypes per 10 μg/m^3^ increase in long-term PM_2.5_ concentrations. The residential areas were considered as a random effect in models (according to the census blocks). The census blocks were randomly selected from each county with a probability of selection proportional to the expected number of households, which were divided into approximately 1000 households. Nearly 5–10% of the households were randomly selected for enumeration in each cluster.

Three models were applied to explore the relationship between long-term exposure to PM_2.5_ and CVD incidence or all-cause mortality including progressively adjusted covariates that were measured at baseline.

Model 1 incorporates age, gender, geographic area (urban or rural), and residential areas as random effects.

Model 2 is inclusive of individual risk factors namely smoking status, physical activity, dietary behavior, obesity, Social Economic Status (SES), and hypertension.

Model 3 is further adjusted in terms of diabetes, total cholesterol, triglycerides, LDL-to-HDL ratio, fatality, and history of heart disease in the family. This model can also be run by residence clustering as random effect.

To investigate individual covariates in association with total CVD, fatal CVD and stroke or AMI as two important subcategories of CVD, the two following hierarchical cox models were implemented.

As a basic model, Model 1 includes each covariate. Model 2 is adjusted by all the remaining covariates and residence clusters as random effect. The *p*-values presented in these models are the interaction results between PM_2.5_ exposure and individual/clinical variables.

The R 4.0.3 software was used to conduct the required analysis.

## Results

Over a 15-year course of following up with 3081 patients, 241 deaths occurred among which 105 death cases resulted from total CVD (fatal AMI, fatal stroke, and SCD). Also, there were 336 non-fatal CVD events (non-fatal incidence of AMIs and strokes). The mean (SD) three-year PM_2.5_ concentration in the study was 45.28 μg/m^3^ (11.63), ranging from a minimum of 20.01 μg/m^3^ in Najafabad to a maximum of 69.80 μg/m^3^ in Isfahan. There was a substantial variation in some individual/clinical variables by PM_2·5_ levels (Table 1). For example, 2429 (78.8%) of the study participants were urban dwellers, among whom 983 (95.8%) were exposed to the lowest PM_2·5_ tertiles, 825 (80.4%) to the middle, and 621(60.3%) to the highest PM_2·5_. In addition, the difference between ever smokers and cholesterol variables in different tertiles was significant, while the difference between the other variables in different tertiles was insignificant.

**Table 1:** Summary of individual characteristics for the 3081 participants across long-term PM_2.5_ exposure tertiles

	All Study Participants	PM_**2·5**_ (μg/m^**3**^) Tertiles			***P***-value
		T1 (< 38.2041)	T2 (38.2041-51.6069)	T3 (> 51.6069)	
No. Individuals	3081	1026	1026	1029	
No. Clusters	37	26	35	32	
No. Events^a^	1060	307	300	453	
Age $\left(\overline{\mathrm{x}},\mathrm{sd}\right)$	49.55 (11.57)	50.11 (11.72)	49.56 (11.54)	48.99 (11.43)	0.090
Female (%)	1589 (51.6)	556 (54.2)	517 (50.4)	516 (50.1)	0.121
Low SES (%)	1045 (33.9)	323 (31.5)	344 (33.5)	378 (36.7)	0.079
Ever Smoker (%)	517 (16.8)	144 (14.0)	188 (18.3)	185 (18.0)	0.049
Low Physical Activity (%)	772 (25.1)	267 (26.0)	270 (26.3)	235 (22.8)	0.373
Poor diet (%)	296 (9.6)	109 (10.6)	89 (8.7)	98 (9.5)	0.320
BMI					0.399
Overweight^b^ (%)	1223 (40.7)	425 (42.2)	387 (38.8)	411 (41.0)	
Obesity^c^ (%)	774 (25.7)	260 (25.8)	270 (27.1)	244 (24.3)	
History of heart disease in the family (%)	162(5.3)	52(5.1)	45(4.4)	65(6.3)	0.138
Hypertension^d^ (%)	771(25.0)	266(25.9)	238(23.2)	267(25.9)	0.254
Diabetes (%)	233(7.6)	80(7.8)	84(8.3)	69(6.8)	0.422
Cholesterol ($\overline{\mathrm{x}},\mathrm{sd}$)	217.85(50.22)	215.80(48.71)	221.63(49.11)	216.16(52.56)	0.014
Triglycerides ($\overline{\mathrm{x}},\mathrm{sd}$)	203.82(124.14)	204.53(127.50)	202.15(128.60)	204.76(116.11)	0.872
Normal LDL to HDL ratio (%)	2710(97.6)	901(97.8)	904(97.1)	905(97.7)	0.425
Urban (%)	2429(78.8)	983(95.8)	825(80.4)	621(60.3)	< 0.001

^a^Fatal CVD(*n* = 105) + Major CVD Events(*n* = 441) + AMI(*n* = 73) + Stroke(*n* = 92) + IHD(*n* = 349)

^b^25 ≤ Body Mass Index< 29.9 ${\mathrm{kg}}\left/{\mathrm{m}^2}\right.$

^c^Body Mass Index≥30 ${\mathrm{kg}}\left/{\mathrm{m}^2}\right.$

^d^Diastolic Blood Pressure > 90 mmHg or Systolic Blood Pressure > 140 mmHg or if the patients were receiving antihypertensive drugs

Model results for all-cause mortality and incidence related to CVDs are presented in Table 2. In Model 1, 10 μg/m^3^ increase in PM_2.5_ was significantly associated with 1.024 HR (0.95 CI = 1.015, 1.033) for CVD, which increased to 1.025 (0.95 CI = 1.016, 1.034) in Model 2. When the other variables were added (Model 3), the HR value increased to 1.026 (0.95 CI = 1.016, 1.036) for CVD events. In the fully adjusted models (Model 3), the most significant association was observed for non-fatal CVD with an HR of 1.029 (0.95 CI = 1.015, 1.034), for AMIs with an HR of 1.031 (0.95 CI = 1.005, 1.057), and for IHDs with an HR of 1.028 (0.95 CI = 1.017, 1.039). No risk association was observed between PM_2.5_ and fatal CVD.

**Table 2:** Adjusted CVD events hazard ratios associated with a 10 μg/m^3^ change in PM_2.5_

	n	Events	Model 1	Model 2	Model 3
**CVD Events**^**a**^	3081	441	1.024 *(1.015,1.033)	1.025 *(1.016,1.034)	1.026 *(1.016,1.036)
**Fatal CVD**^**b**^	3081	105	0.995 (0.978,1.013)	0.955 (0.977,1.012)	0.998 (0.979,1.017)
**None-fatal CVD**	3081	336	1.025 *(1.010,1.032)	1.028 *(1.011,1.032)	1.029 *(1.015,1.034)
**All-cause mortality**^**c**^	3081	241	0.974 (0.961,0.987)	0.972 (0.959,0.985)	0.974 (0.960,0.988)
**AMI**^**d**^	3081	73	1.019 (0.997,1.041)	1.019 (0.997,1.041)	1.031 *(1.005,1.057)
**Stroke**	3081	92	1.018 (0.999,1.037)	1.017 (0.999,1.036)	1.018 (0.999,1.039)
**IHD**^**e**^	3081	349	1.026 *(1.016,1.037)	1.027 *(1.017,1.038)	1.028 *(1.017,1.039)

Model 1: PM_2.5_, age, sex, urban/rural status and cluster random effect

Model 2: PM_2.5_, age, sex, smoking status, physical activity, dietary behaviors, obesity, SES, hypertension status, urban/rural status and cluster random effect

Model 3: PM_2.5_, age, sex, smoking status, physical activity, dietary behaviors, obesity, SES, hypertension status, diabetes, cholesterol, triglycerides, LDL to HDL ratio, history of heart disease in the family, urban/rural status, and cluster random effect

* Statistically significant (0.05)

^a^CHD + stroke

^b^Death from cardiovascular causes and myocardial infarction, stroke, and heart failure, with each sub-category including fatal events

^c^All of the deaths that occur in a population, regardless of the cause

^d^Acute myocardial infarction

^e^MI+ SCD + UAP

The relationships of PM_2.5_ with fatal CVD, CVD, AMI, and stroke events according to the individual/clinical variables are summarized in Tables 3 and 4. The results illustrate that the effect of heterogeneity (or interaction with PM_2.5_) concerning different ages, gender, smoking status, high blood pressure, and diabetes status was statistically significant for different events.

**Table 3:** Estimated HRs for Fatal CVD, CVD AMI, and stroke events associated with an increase of 10 μg/m^3^ in PM_2.5_, according to individual and clinical variables

Characteristic	Fatal CVD			CVD Events			AMI Events			Stroke Events		
	HR (95% CI)^**a**^	HR (95% CI)^**b**^	***P***^**c**^	HR (95% CI)^**a**^	HR (95% CI)^**b**^	***P***^**c**^	HR (95% CI)^**a**^	HR (95% CI)^**b**^	***P***^**c**^	HR (95% CI)^**a**^	HR (95% CI)^**b**^	***P***^**c**^
**Gender**			0.750			0.001			0.125			0.171
**Female**	Ref.	Ref.		Ref.	Ref.		Ref.	Ref.		Ref.	Ref.	
**Male**	1.712 (1.020,2.873)	1.009 (0.998,1.020)		1.351 (1.051,1.735)	1.006 (1.001,1.011)		2.167 (1.148,4.090)	1.013 (1.000,1.027)		1.153(0.678,1.958)	1.003 (0.992,1.014)	
**Age**			0.050			0.004			0.078			0.001
**< 60**	Ref.	Ref.		Ref.	Ref.		Ref.	Ref.		Ref.	Ref.	
**> 60**	6.943 (4.182,11.527)	1.046 (1.033,1.060)		2.524 (2.005,3.177)	1.017 (1.012,1.022)		1.903 (1.045,3.468)	1.009 (0.996,1.022)		4.687 (2.880,7.628)	1.031 (1.020,1.043)	
**SES**	0.998 (0.990,1.001)	0.999 (0.989,1.011)	0.258	0.997 (0.980,1.010)	0.999 (0.994,1.003)	0.780	0.997 (0.991,1.012)	1.000 (0.988,1.013)	0.138	1.001 (0.988,1.014)	0.996 (0.986,1.007)	0.700
**Smoking Status**			0.935			0.03						0.913
**None**	Ref.	Ref.		Ref.	Ref.		Ref.	Ref.		Ref.	Ref.	
**Ever**	1.336 (0.803,2.221)	1.004 (0.993,1.016)		1.411 (1.07,1.817)	1.006 (1.001,1.011)		1.410 (0.755,2.634)	1.009 (0.997,1.022)		1.577(0.926,2.687)	1.007 (0.996,1.018)	
**Physical Activity**	0.402 (0.193,0.837)	0.991 (0.984,0.998)	< 0.001	0.627 (0.457,0.859)	0.995 (0.992,0.998)	0.001	0.402 (0.157,1.032)	0.992 (0.984,0.999)	0.004	0.435 (0.205,0.920)	0.993 (0.986,1.000)	0.03
**Dietary behaviors**			0.515			0.816			0.179			0.474
**Poor**	Ref.	Ref.		Ref.	Ref.		Ref.	Ref.		Ref.	Ref.	
**Healthy**	1.104 (0.551,2.211)	1.004 (0.988,1.011)		1.087(0.778,1.520)	1.001 (0.994,1.008)		0.692 (0.324,1.478)	0.992 (0.977,1.007)		1.067 (0.530,2.148)	1.001 (0.986, 1.016)	
**Hypertension Status**			0.001			0.001			0.117			< 0.001
**No**	Ref.	Ref.		Ref.	Ref.		Ref.	Ref.		Ref.	Ref.	
**Yes**	1.809 (1.161,2.818)	1.013 (1.003,1.023)		1.783 (1.431,2.223)	1.012 (1.007,1.016)		1.394 (0.782,2.485)	1.007 (0.996,1.019)		1.924 (1.210,3.059)	1.012 (1.002,1.022)	
**Diabetes**			0.020			0.040			0.010			0.010
**No**	Ref.	Ref.		Ref.	Ref.		Ref.	Ref.		Ref.	Ref.	
**Yes**	3.964 (2.437,6.448)	1.033 (1.022,1.044)		2.238 (1.675,2.991)	1.016 (1.009,1.022)		3.650 (1.877,7.099)	1.025 (1.011,1.039)		3.255 (1.888,5.611)	1.024 (1.012,1.037)	
**Cholesterol**	1.003 (0.998,1.008)	1.000 (1.000,1.000)	0.867	1.002 (1.000,1.005)	1.000 (1.000,1.000)	0.005	1.010 (1.005,1.015)	1.000 (1.000,1.000)	0.020	0.997 (0.992,1.002)	1.000 (1.000,1.000)	0.368
**Triglycerides**	0.997 (0.994,1.001)	1.000 (0.999,1.000)	0.116	1.000 (0.999,1.002)	1.000 (1.000,1.000)	0.687	0.998 (0.994,1.001)	1.000 (0.999,1.000)	0.811	1.000 (0.997,1.003)	1.000 (1.000,1.000)	0.146
**LDL to HDL ratio**	0.145 (0.016,1.319)	0.981 (0.965,0.998)	0.003	1.252 (0.170,9.253)	0.998 (0.988,1.008)	0.638	–	1.021 (0.988,1.056)	0.362	–	–	–
**BMI**	0.865 (0.478,1.563)	0.996 (0.983,1.009)	0.695	1.252 (0.170,9.253)	1.002 (0.997,1.007)	0.375	1.022 (0.529,1.974)	1.000 (0.9987,1.013)	0.346	1.508 (0.902,2.520)	1.010 (1.000,1.021)	0.910
**History of heart disease in the family**			0.501			0.714			0.097			0.344
**No**	Ref.	Ref.		Ref.	Ref.		Ref.	Ref.		Ref.	Ref.	
**Yes**	1.133 (0.540,2.378)	1.003 (0.987,1.018)		1.109 (0.754,1.630)	1.002 (0.994,1.009)		1.037 (0.367,2.925)	0.999 (0.980,1.020)		0.978 (0.420,2.280)	1.000 (0.984,1.018)	
**Urban/Rural status**			0.117			0.001			0.004			0.004
**Urban**	Ref.	Ref.		Ref.	Ref.		Ref.	Ref.		Ref.	Ref.	
**Rural**	0.988 (0.971,1.001)	0.987 (0.973,1.000)		0.988 (0.975,1.011)	0.989 (0.984,0.994)		0.988 (0.975,1.011)	0.984 (0.970,0.998)		0.986 (0.970,1.000)	0.987 (0.975,1.000)	

^a^Crude model: adjusted no factors associated with CVD events

^b^Adjusted model: adjusted for gender (coded as female/male), age (coded as < 60/> 60), SES (continues), smoking status (coded as none/ever), physical activity (continues), dietary behaviors (coded as poor/healthy), hypertension status (coded as no/yes), diabetes (coded as no/yes), cholesterol (continues), triglycerides (continues), LDL to HDL ratio (continues), BMI (continues), history of heart disease in the family (coded as no/yes), urban/rural status (coded as urban/rural), cluster random effect

^c^*P*-values are the interaction results between PM_2.5_ exposure and individual/clinical variables

**Table 4:** Estimated HRs for fatal CVD and CVD events associated with PM_2.5_ tertiles, according to individual and clinical variables

Characteristic	Fatal CVD			CVD Events			AMI Events			Stroke Events		
	HR (95% CI)^**a**^	HR (95% CI)^**b**^	***P***^**c**^	HR (95% CI)^**a**^	HR (95% CI)^**b**^	***P***^**c**^	HR (95% CI)^**a**^	HR (95% CI)^**b**^	***P***^**c**^	HR (95% CI)^**a**^	HR (95% CI)^**b**^	***P***^**c**^
**Gender**			0.002			0.001			0.073			0.527
**Female**	Ref.	Ref.		Ref.	Ref.		Ref.	Ref.		Ref.	Ref.	
**Male**	1.795 (1.068,3.018)	1.265 (1.003,1.595)		1.394 (1.084,1.793)	1.152 (1.032,1.286)		2.242 (1.185,4.242)	1.302 (0.976,1.737)		1.208 (0.709,2.058)	1.108 (0.881,1.394)	
**Age**			< 0.001			0.035			0.207			< 0.001
**< 60**	Ref.	Ref.		Ref.	Ref.		Ref.	Ref.		Ref.	Ref.	
**> 60**	6.995 (4.218,11.601)	2.606 (1.956,3.471)		2.497 (1.984,3.142)	1.416 (1.274,1.581)		1.871 (1.027,3.409)	1.194 (0.902,1.580)		4.624 (2.844,7.518)	1.869 (1.474,2.370)	
**SES**	0.971 (0.882,1011)	0.970 (0.763,1.234)	0.451	0.988 (0.880,1.023)	0.981 (0.884,1.089)	0.999	0.849 (0.664,1.211)	0.984 (0.751,1.290)	0.525	0.861 (0.724, 1.171)	0.922 (0.737,1.154)	0.283
**Smoking Status**			0.770			0.078						0.316
**None**	Ref.	Ref.		Ref.	Ref.		Ref.	Ref.		Ref.	Ref.	
**Ever**	1.299 (0.780,2.162)	1.004 (0.787,1.279)		1.401(1.089,1.802)	1.110 (0.996,1.237)		1.407 (0.753,2.629)	1.209 (0.922,1.584)		1.536 (0.902,2.615)	1.119 (0.888,1.409)	
**Physical Activity**	0.387 (0.186,0.808)	0.834 (0.717,0.967)	0.040	0.612 (0.446,0.840)	0.896 (0.838,0.958)	0.044	0.389 (0.151,0.999)	0.813 (0.678,0.976)	0.046	0.421 (0.199,0.892)	0.855 (0.739,0.991)	0.04
**Dietary behaviors**			0.120						0.096			0.231
**Poor**	Ref.	Ref.		Ref.	Ref.		Ref.	Ref.		Ref.	Ref.	
**Healthy**	1.101 (0.549,2.207)	1.091 (0.768,1.549)		1.091(0.780,1.525)	1.016 (0.878,1.174)		0.697 (0.327,1.489)	0.862 (0.612,1.215)		1.063 (0.528,2.138)	0.994 (0.732,1.351)	
**Hypertension Status**			0.001			< 0.001			0.209			0.023
**No**	Ref.	Ref.		Ref.	Ref.		Ref.	Ref.		Ref.	Ref.	
**Yes**	1.832 (1.177,2.853)	1.321 (1.074,1.626)		1.754 (1.407,2.185)	1.246 (1.132,1.371)		1.376 (0.773,2.448)	1.144 (0.890,1.470)		1.914 (1.206,3.039)	1.278 (1.043,1.565)	
**Diabetes**			0.021			0.002			0.005			0.001
**No**	Ref.	Ref.		Ref.	Ref.		Ref.	Ref.		Ref.	Ref.	
**Yes**	4.179 (2.555,6.833)	2.003 (1.586,2.530)		2.278 (1.705,3.045)	1.418 (1.247,1.613)		3.688 (1.897,7.169)	1.664 (1.231,2.249)		3.375 (1.952,5.838)	1.693 (1.316,2.177)	
**Cholesterol**	1.003 (0.998,1.009)	1.002 (0.999,1.004)	0.112	1.003 (1.000,1.005)	1.001 (1.000,1.002)	0.042	1.010 (1.005,1.015)	1.004 (1.002,1.006)	0.041	0.997 (0.992,1.002)	0.999 (0.997,1.002)	0.705
**Triglycerides**	0.997 (0. 994,1.000)	0.999 (0.997,1.000)	0.152	1.000 (0.999,1.002)	1.000 (0.999,1.000)	0.060	0.998 (0.994,1.001)	0.999 (0.998,1.01)	0.808	1.000 (0.997,1.003)	0.999 (0.998,1.001)	0.421
**LDL to HDL ratio**	0.184 (0.020,1.708)	0.683 (0.481,0.971)	0.245	1.190 (0.161,8.778)	0.964 (0.782,1.188)	0.147	–	1.597 (0.728,3.508)	0.599	–	–	–
**BMI**	0.904 (0.498,1.638)	0.993 (0.748,1.319)	0.781	1.141 (0.889,1.465)	1.069 (0.959,1.191)	0.240	1.027 (0.531,1.988)	0.990 (0.745,1.316)	0.782	10,541 (0.921,2.580)	1.303 (1.042,1.628)	< 0.001
**History of heart disease in the family**			0.248			0.326			0.099			0.084
**No**	Ref.	Ref.		Ref.	Ref.		Ref.	Ref.		Ref.	Ref.	
**Yes**	1.066 (0.507,2.242)	0.946 (0.671,1.334)		1.100 (0.748,1.618)	1.022 (0.870,1.202)		1.044 (0.371,2.942)	0.999 (0.646,1.543)		0.965 (0.415,2.247)	1.021 (0.717,1.453)	
**Urban/Rural status**			0.012			0.038			0.043			0.035
**Urban**	Ref.	Ref.		Ref.	Ref.		Ref.	Ref.		Ref.	Ref.	
**Rural**	0.751 (0.513,0.957)	0.684 (0.512,0.915)		0.841 (0.658,0.954)	0.768 (0.686,0.858)		0.548 (0.311, 0.867)	0.680 (0.493,0.937)		0.858 (0.750,.970)	0.733 (0.570,0.944)	

^a^Crude model: adjusted no factors associated with CVD events

^b^Adjusted model: adjusted for gender (coded as female/male), age (coded as < 60/> 60), SES (continues), smoking status (coded as none/ever), physical activity (continues), dietary behaviors (coded as poor/healthy), hypertension status (coded as no/yes), diabetes (coded as no/yes), cholesterol (continues), triglycerides (continues), LDL to HDL ratio (continues), BMI (continues), history of heart disease in the family (coded as no/yes), urban/rural status (coded as urban/rural), and cluster random effect

^c^*P*-values are the interaction results between PM_2.5_ tertiles exposure and individual/clinical variables

For example, per 10 μg/m^3^ increase in PM_2·5_ for those subjects in the age of > 60 years, the HR was 1.046 for fatal CVD, 1.017 for CVD events, and 1.031 for stroke events. Moreover, the male gender determined HR of 1.006 for CVD events. Ever-smokers were subject to HR of 1.006 for CVD events and those with diabetes were subject to HR of 1.033 for fatal CVD, 1.016 for CVD events, 1.025 for AMI events, and 1.024 for stroke events (P for heterogeneity < 0·05). Overall, the results in Table 3 suggest that the effect of PM_2.5_ on cardiovascular disease was stronger among older people, smokers, and those with high blood pressure and diabetes.

According to the tertiles models, increasing the risk of CVD and stroke events was presented only for PM_2.5_ > 51.61 μg/m^3^.

**Fig. 1:**
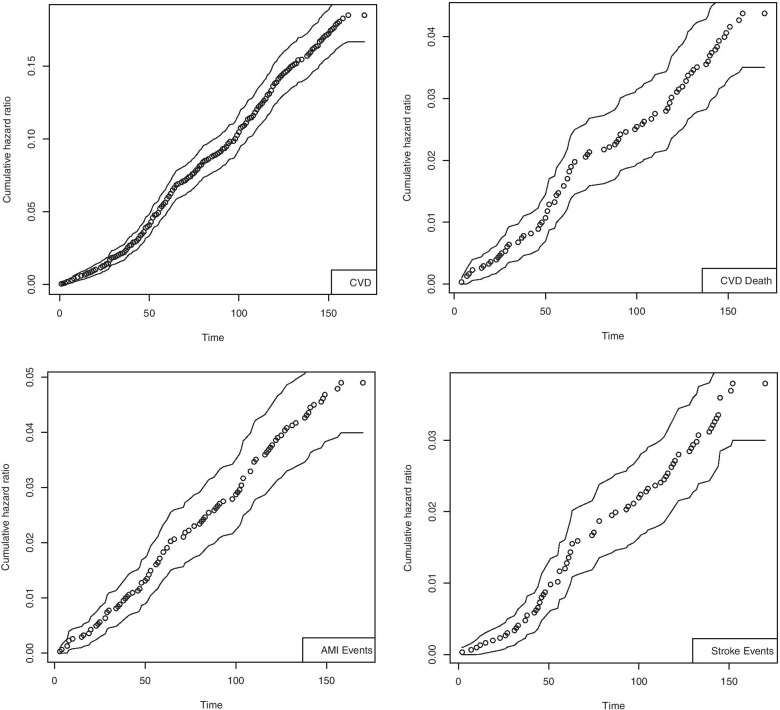
Cumulative hazard estimates with 95% confidence interval for different events (Model 3)

Figure 1 shows the cumulative risk ratio and its associated confidence interval for different outcomes, based on which an upward trend could be perceived.

## Discussion

In Isfahan province, long-term outdoor PM_2·5_ exposure results from major CVD events. The findings of the present study offer new information on the relationship between ambient PM_2·5_ exposure and both CVD incidence and mortality in a wide range of PM_2.5_ concentrations (20.01 to 69.80 μg/m^3^) in different urban/rural areas while adjusting a comprehensive set of individual confounders.

Particulate Matter is of particular importance due to its specific characteristics such as composition and size distribution. This pollutant is characterized by a very large surface area and it could be adsorbed by many diverse organic materials such as polycyclic aromatic hydrocarbons, nitro-polycyclic aromatic hydrocarbons, heavy metals, pathogens, and radioactive materials. It contains fine particles penetrating the lower respiratory system and the blood and migrating to other organs, even to the brain [36].

According to the WHO recommendation, long-term exposure to extreme levels of PM_2.5_ over 10-25 μg/m^3^ could potentially impair the coagulation process, reduce inflammation, damage blood vessels, and eventually cause cardiovascular disease. Several pathways that could help justify the strong link between PM_2.5_ and cardiovascular diseases were identified [37, 38].

In Europe (Barcelona), PM_2.5_ concentration in the air of urban areas has been decreasing, and reducing air pollution in the metropolitan area of Barcelona would bring about substantial health and economic benefits [39].

In the present study, data sets obtained from environmental monitoring stations specific to a limited time period were provided and the correlation between satellite information and the data sets was found reasonable (R^2^ > 0.60).

Similar to our study, other researchers have utilized satellite data to measure air pollution and examine the association between these data and cardiovascular diseases [40–43].

A research study was conducted in Tehran to investigate the impact of temporal changes on the concentration of PM_2.5_ and a wide range of diseases. Reportedly, long-term exposure to PM_2.5_ caused deaths, estimated to be ranging between 24.5 and 36.2% out of total deaths induced by cerebrovascular diseases (stroke) and between 19.8 and 24.1% by IHD. They also found that IHD-induced deaths constituted the largest number of deaths resulting from long-term exposure to PM_2.5_. Overall, long-term exposure to PM_2.5_ significantly contributes to mortality in Tehran [44]. Our results are consistent with those reported in this Iranian study, although we have found the significant effects only in the case of CVD incidence rather than CVD mortality [44].

Chen et al. (2008) conducted a systematic review of the relationship between long-term exposure to ambient pollution and chronic diseases. They found that such PM_2·5_ exposure would increase the risk of cardiovascular mortality by nearly 12–14% per 10-μg/m^3^ increase in PM_2·5_, independent of age, gender, and geographic region [45]. Our results for CVD-related deaths turned out to be insignificant statistically due to the smaller sample size as compared to the results of different reviewed studies [45].

Inconsistency was found between our study and other recently conducted meta-analyses in which 53 studies on long-term PM_2.5_ and mortality in more than 150,000 adults with different SES levels were reviewed [46]. It was demonstrated that in the case of all-cause mortality, a 1 μg/m^3^ increase in PM_2.5_ was significantly associated with a 1.29% increase in all-age and all-cause mortality (95%CI 1.09-1.50) at a mean exposure of 10 μg/m^3^. The percentage of increase in the rate of cardiovascular mortality was significant at 1.46% (95% CI, 1.25–1.67) with a mean exposure of 10 μg/m^3^.

Moreover, our study observed that the mean (SD) of 3-year PM_2·5_ concentrations was 45.28 (11.63) μg/m^3^ with the HR value of 1.026 (0.95 CI = 1.016, 1.036) per 10 μg/m^3^ increase in PM_2·5_ for all CVD events (see Table 2). Of note, Model 3 is a complete model that considers both confounders adjustment and residence clustering as a random effect. These estimates were considerably smaller than those cited in the meta-regression analyses due to the smaller sample size of our study in comparison to the result of different reviewed studies. However, some researchers have employed different modeling approaches (e.g., space-time exposure models) instead of land use regression method or fixed monitoring stations and considered the zip-codes of their study subjects controlling for SES levels; therefore, their approach estimated a larger number of deaths, unlike our approach which estimated a lower rate of mortality due, mainly, to the smaller sample size [46].

Outcome of the adjusted models achieved by Hystad et al. [12] demonstrated that during a median follow-up period of 9·3 years (between Jan 1, 2003 and July 14, 2018) targeting 157,436 adults belonging to 747 communities in 21 countries, a 10 μg/m^3^ increase in PM_2·5_ was associated with an increased risk for cardiovascular disease events (hazard ratio 1·05 [95% CI 1·03–1·07]), myocardial infarction (1·03 [1·00–1·05]), stroke (1·07 [1·04–1·10]), and cardiovascular disease mortality (1·03 [1·00–1·05]). They concluded that long-term outdoor PM_2·5_ concentrations were associated with increased risks of cardiovascular disease in adults aging 35–70 years. In terms of the risk of contracting cardiovascular disease, our research shares similarities to the above-mentioned research study.

In another study carried out by Abdolahnejad et al., Relative Risk (RR) pointed to the increased risk of exposure to pollutants, as obtained through time-series experiments. In Isfahan, upon every 10 μg/m^3^ increase in the concentration of the pollutant, the value of RR per increase in the total mortality induced by PM_10_ was 0.8% [47]. Our results are inconsistent with those of Abdolahnejad et al. and this divergence results from different modeling methods and follow-up periods.

Hvidtfeldt UA et al. put a greater emphasis on exposure data from central air monitoring stations and followed a Danish cohort of 49,564 individuals from the years 1993–1997 to 2015. Among the 895,897 annual follow-up cases, 10,193 deaths induced by all causes occurred, of which 2319 were CVD related. They observed that the relationship between long-term exposure to PM_2.5_ and CVD mortality was significant (hazard ratio of 1.03; 95% confidence interval: 1.01, 1.05) [48]. Our results were not significant because of the longer follow-up period and the larger number of participants [48].

Zhang et al. investigated a retrospective cohort consisting of 39,054 subjects from four cities in northern China for the mortality induced by all-cause and specific cardiovascular diseases over the course of the years 1998 to 2009 in order to assess the cardiovascular effects of long-term exposure to high-level concentrations of inhalable particulate. Their analyses suggested that the effects of PM_10_ on cardiovascular mortality were more pronounced in males, smokers, and people with a higher socioeconomic status. Long-term exposure to PM_10_ increases mortality from cardiovascular disease, especially from ischemic heart disease [49]; this finding is consistent with our presentation.

Chen et al. performed a random-effects meta-analysis. This study included cohort and case-control studies on outdoor air pollution in human populations using individual-level data. In addition to natural-cause mortality, they evaluated mortality from circulatory diseases (IHD and cerebrovascular disease (stroke)). They concluded that there was enough evidence to suggest that both PM_2.5_ and PM_10_ were associated with increased mortality from all causes and cardiovascular disease [50]. This result is not in agreement with that of our study because the aforementioned research study carried out different types of outdoor exposure and checked PM_2.5_ and PM_10_ simultaneously [50].

In the present paper, following each 10 μg/m^3^ increase in PM_2.5_, HR for fatal CVD was measured at 0.998 (0.95 CI = 0.979, 1.017). Therefore, no evidence of risk associations was observed between PM_2.5_ increase and fatal CVD. Findings acquired from Tseng et al. confirmed the results of the present study. A cohort study was carried out on the potential associations between PM_2.5_ and cardiovascular mortality with 43,227 patients in Taiwan. Participants were followed up from the year 1989 to 2008 and their vital status was determined based on death records. In this study, no evidence of an increased risk of all-cause or cardiovascular mortality with a prolonged exposure to PM_2.5_ was detected [51], similar to our study.

Regional characteristics of Isfahan are the reasons for a higher percentage of PM_2.5_ concentration in nearby rural areas. The occurrence of this phenomenon could be attributed to the proximity of these areas to deserts and swamps. Deserts are severely affected by wind erosion and due to the prevailing wind direction in these areas blowing from the east to the west, many suspended particles are transported to the western side, inevitably causing air pollution. The Gavkhouni Wetland, i.e., the endpoint of Zayanderud (a famous river in Isfahan) which is a place to trap suspended particles, has run dry due to reduced rainfall and improper farming and irrigation methods and has turned into a source of many dust particles in the studied period.

Furthermore, sand mines and brick kilns situated in the east and northeast of Isfahan are essential sources of the production of dust particles and their spread to the air of the city. Similarly, anomalies could be observed as the average concentration of suspended particles in different city areas. Such anomalies could be caused by various factors such as industrial activities in the suburbs, vehicle and urban traffic, proximity of the area to the landfill, reduced vegetation, and green spaces [52, 53].

The main objective of the study of Khusfi et al. was to investigate the effects of atmospheric conditions and physical characteristics of the Earth on the seasonal variations in dust concentration in the semi-arid regions of Central Iran Zone (CIZ) in a typical period (2001–2008 and 2009–2016). It was found that the activity of Sand and Dust Storms (SDS) in 2009-2016 was greater than that in the previous period (2001-2008), i.e., a finding consistent with our study results [54].

Indeed, according to the results presented in Table 1, 78.8% of the cohort population inhabited the urban areas. However, in the third tertile, this number was 60.3% (urban areas in the third tertile of PM_2.5_ decreased in number and more rural areas were included), and the difference between the two mentioned values was significant [52, 53].

Moreover, in rural areas, villagers are exposed to smoke from burning agricultural waste. These results were similarly obtained using a cross-sectional study performed through random selection of three villages in Isfahan in order to evaluate the respiratory effects of this smoke-induced air pollution. It was shown that in symptomatic cases before and after rice burning, the total particulate matters doubled during the burning time. In this study, all clinical and spirometry changes were statistically significant between study subjects [55].

Based on our results, only CVD events and stroke, as its essential subtype, had a significantly strong dose-response relationship; HRs related to the 3rd tertile of PM_2·5_ were1.652 (0.95 CI = 1.269, 2.150) for total CVD events and 1.791 (0.95 CI = 1.036, 3.097) for stroke events. Taken together, our study results support the evidence of a significant increase in total CVD risk, especially for stroke, at high PM_2·5_ concentrations.

In addition to the diverse geographical population and extensive PM concentration range, the ICS is unique in its depth of individual variables to adjust the potential confounding factors.

Besides the long-term follow-up (15 years), ICS considered a range of sociodemographic, behavioral, metabolic, and clinical variables that affect CVD, an issue that most large cohort studies examining air pollution did not measure [52].

From the results, one could conclude that long-term outdoor PM_2·5_ is a significant contributing factor in CVD in Iran. While it is unlikely that outdoor PM_2·5_ concentrations would vary substantially over such small areas, some exposure misclassifications exist that could bias estimates towards the null.

We compared PM_2.5_ concentrations and CVD events across different areas. While residual confounding cannot be ruled out, we adjusted more individual CVD risk factors than previous studies; the analyses controlling for unmeasured factors among the centers using random-effects demonstrated larger estimates of the effect. We could not examine specific causes of non-fatal CVD due to the smaller number of events; however, these analyses can be done in the future with additional follow-ups and more events.

The present research enjoys some merits including being a longitudinal study with a 15-year follow-up, sufficient number of samples, inclusive outcomes, sufficient number of urban and rural population exposed to high PM_2.5_ concentrations for the whole study period, objective measurement of a comprehensive suite of individual CVD risk factors, standardized collection of data on household and community characteristics, and prospective recording of fatal and non-fatal events that were evaluated through standard definitions [15, 34, 35].

The present study was subject to the following limitations. First, there was an insufficient scope data on indoor air pollution sources such as household use of solid fuels. Second, exposure to ambient PM_2.5_ was estimated based on the participants’ residential addresses regardless of their daily activities and location changes during days. Third, acute (i.e., daily) variations in PM_2.5_ exposures and their impacts could not be controlled. Fourth, we did not consider local environmental monitoring data because our study was a 15-year cohort. Existing local environmental monitoring data for these 15 years were not entirely available; local environmental monitoring centers were limited; and the distance from the patients’ residential neighborhoods was far away from the stations. However, satellite data with a 1 × 1 km spatial resolution included the patients’ residential addresses which were more accurate while local environmental monitoring data were not satisfactory in terms of quality.

## Conclusions

The present study demonstrated that long-term ambient PM_2.5_ exposure was associated with the increased risk of contracting CVDs including AMI, IHD, and strokes. Our data support the assumption that PM_2.5_ is an important risk factor in CVD.

## Supplementary Information

**Additional file 1: Table 1.** The local seasons, based on the local climate.

**Additional file 2: Table 2.** The results of PM_2.5_-AOD seasonal regressions.

## Data Availability

The datasets generated and/or analyzed during the current study are available upon request to the corresponding author.

## Abbreviations

PM_2.5_: Particles with a mass median aerodynamic diameter ≤ 2.5 μm
CVD: Cardio-Vascular Disease
AMI: Acute Myocardial Infarction
IHD: Ischemic heart disease
CHD: Coronary heart disease
MI: Myocardial infarction
SCD: Sudden cardiac death
UAP: Unstable angina pectoris
SES: Social Economic Status
HR: Hazard Ratio
