# Supplementary material for: Long-term exposure to PM2.5 and cardiovascular disease incidence and mortality in an Eastern Mediterranean country: findings based on a 15-year cohort study
Source: Environ Health. 2021 Oct 28;20:112. doi: 10.1186/s12940-021-00797-w (PMC8555193; doi:10.1186/s12940-021-00797-w)
Supplement: Supplementary file 1 — Additional file 1: Table 1. The local seasons, based on the local climate. [file 12940_2021_797_MOESM1_ESM.docx]

Table 1. The local seasons, based on the local climate.

| **Season** | **from** | **to** |
| --- | --- | --- |
| Spring | 1 Mar | 1 Jun |
| Summer | 1 Jun | 10 Sep |
| Autumn | 10 Sep | 1 Dec |
| Winter | 1 Dec | 1 Mar |
