# Supplementary material for: Long-term exposure to PM2.5 and cardiovascular disease incidence and mortality in an Eastern Mediterranean country: findings based on a 15-year cohort study
Source: Environ Health. 2021 Oct 28;20:112. doi: 10.1186/s12940-021-00797-w (PMC8555193; doi:10.1186/s12940-021-00797-w)
Supplement: Supplementary file 2 — Additional file 2: Table 2. The results of PM2.5-AOD seasonal regressions. [file 12940_2021_797_MOESM2_ESM.docx]

Table 2. The results of PM_2.5_-AOD seasonal regressions.

| **Season** | **a** | **b** | **R Square** | **Std. Error of the Regression** | **Statistical test** |
| --- | --- | --- | --- | --- | --- |
| Spring | 185 ± 7.0 | 30 ± 2.0 | 0.62 | 15.2 | passed |
| Summer | 165 ± 6.5 | 31 ± 2.0 | 0.57 | 14.6 | passed |
| Autumn | 250 ± 11.5 | 25 ± 3.0 | 0.52 | 21.3 | passed |
| Winter | 240 ± 10. 0 | 29 ± 2.0 | 0.55 | 16.9 | passed |
